# Potential therapeutic effect of olfactory ensheathing cells in neurological diseases: neurodegenerative diseases and peripheral nerve injuries

**DOI:** 10.3389/fimmu.2023.1280186

**Published:** 2023-10-17

**Authors:** Li-peng Zhang, Jun-xiang Liao, Yi-yi Liu, Hong-lang Luo, Wen-jun Zhang

**Affiliations:** ^1^ Department of Rehabilitation Medicine, The Second Affiliated Hospital, Nanchang University, Nanchang, Jiangxi, China; ^2^ The Second Affiliated hospital, Nanchang University, Nanchang, Jiangxi, China

**Keywords:** OECS, neurological diseases, treatment, nerve regeneration, role

## Abstract

Neurological diseases are destructive, mainly characterized by the failure of endogenous repair, the inability to recover tissue damage, resulting in the increasing loss of cognitive and physical function. Although some clinical drugs can alleviate the progression of these diseases, but they lack therapeutic effect in repairing tissue injury and rebuilding neurological function. More and more studies have shown that cell therapy has made good achievements in the application of nerve injury. Olfactory ensheathing cells (OECs) are a special type of glial cells, which have been proved to play an important role as an alternative therapy for neurological diseases, opening up a new way for the treatment of neurological problems. The functional mechanisms of OECs in the treatment of neurological diseases include neuroprotection, immune regulation, axon regeneration, improvement of nerve injury microenvironment and myelin regeneration, which also include secreted bioactive factors. Therefore, it is of great significance to better understand the mechanism of OECs promoting functional improvement, and to recognize the implementation of these treatments and the effective simulation of nerve injury disorders. In this review, we discuss the function of OECs and their application value in the treatment of neurological diseases, and position OECs as a potential candidate strategy for the treatment of nervous system diseases.

## 1 Introduction

The ability of nerve regeneration and tissue repair is limited after the occurrence of neurological diseases, which can usually lead to permanent disability. Neurological diseases, especially neurodegenerative diseases, describe a clinical condition characterized by selective and progressive neuronal loss. Neurological diseases caused by progressive loss of neuronal structure or function, and lead to varying degrees of paralysis and cognitive and sensory loss (1). Neurological diseases such as Parkinson’s disease (PD), Alzheimer’s disease (AD), Huntington’s disease (HD), and amyotrophic lateral sclerosis (ALS), characterized by progressive loss of the structure, function, or number of neurons in the brain or spinal cord (2, 3). Although the clinical use of some drugs can alleviate the progression of these diseases. New drugs for the treatment of neurological diseases have become urgently needed. Clinicians, patients and their families are waiting for the development of effective drugs for neurological diseases that are basically untreatable at present. The drug development process is complex and expensive, the clinical use of these diseases is limited. Unfortunately, the treatment currently available are not sufficient to prevent neurodegeneration. Therefore, it is necessary to find and explore prospective therapeutic method to repair neurological function and improve symptoms.

With the exploration of treatment methods, researchers have found that cell therapy have developability and broad prospects, and have become the savior of many diseases. The use of cell therapy to treat neurological diseases is based on the assumption that these therapies will mimic the normal process of cell repair and development in the nervous system to eliminate the causes of the disease (4, 5). Cell therapy, also known as regenerative therapy, uses special types of active cells or their derivatives to improve the repair response of dysfunctional and damaged tissue (6, 7). OECs are special glial cells, which can survive and renew in the central nervous system, and have been widely used in nerve regeneration and tissue repair (8, 9). OECs therapy usually focuses on cell substitution or environmental enrichment. Application of OECs therapy provides a valuable and attractive choice for neurological diseases. Transplantation of OECs to the injured nerve can promote regeneration and reconstruct the nerve function (10). The function of OECs in the treatment of neurological diseases includes secreted active biomolecules, such as neurotrophic factors, VEGF, NT-3 and extracellular matrix. The production of these secretions provides a good nutritional basis for axonal regeneration and myelination (11, 12). Moreover, OECs can exert neuroprotection and immune regulation to improve the activity of immune cells in nerve injury, reduce nerve injury and nerve inflammation, and play a therapeutic role (11, 13, 14). All these reflect the potential and sustainability of OECs in the treatment of neurological diseases. Therefore, we focus on the functional role of OECs in neurological diseases and the existing problems as a cell therapy.

## 2 A brief introduction to the biological characteristics of OECs

**Figure 1:**
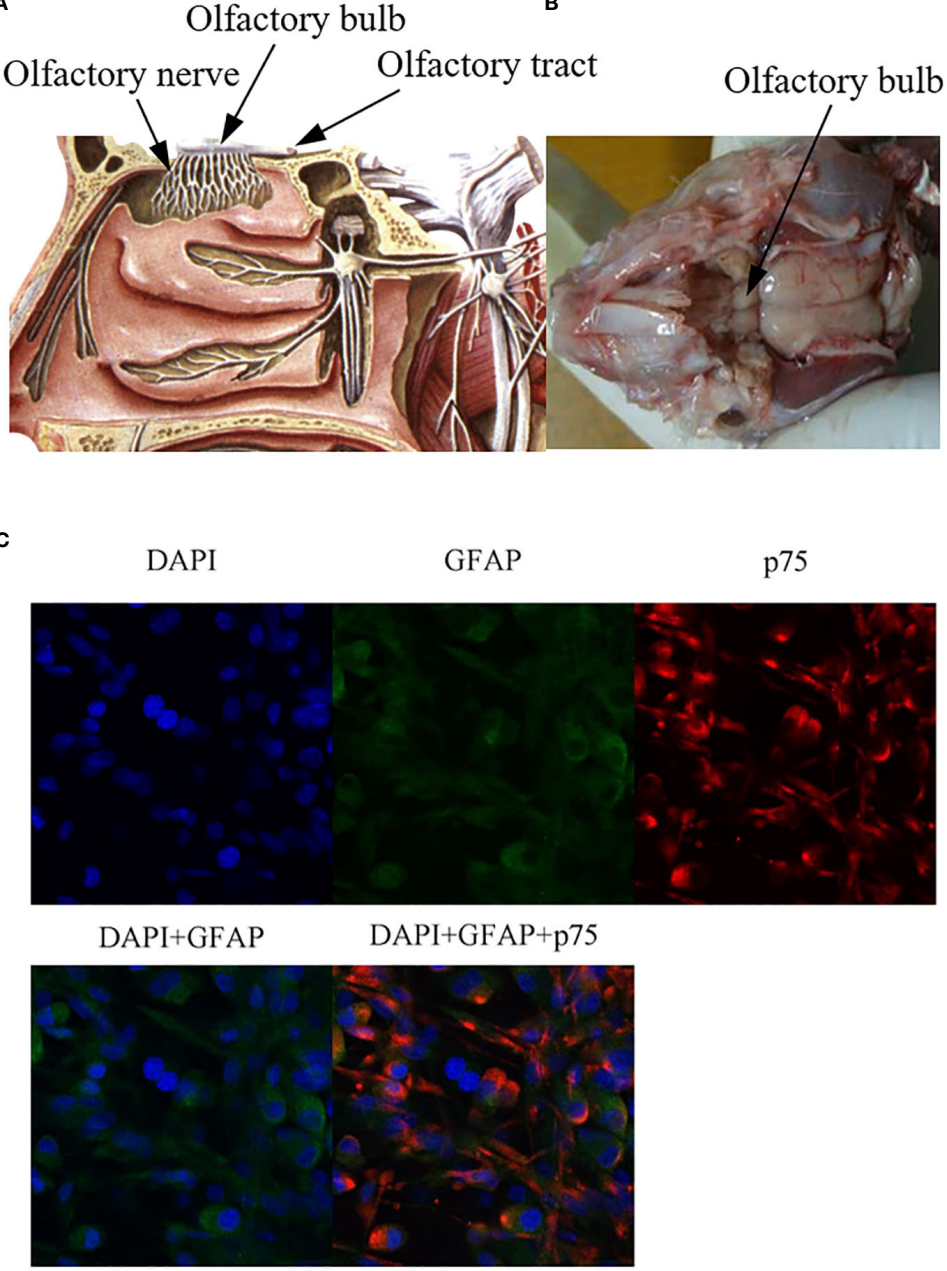
Anatomical localization of olfactory bulb and immunofluorescence staining of OECs. **(A)** Anatomical diagram of olfactory bulb tissue, source site of OECs. **(B)** Schematic diagram of olfactory bulb tissue in rats. Olfactory bulb tissues were lysed and digested *in vitro*, and OECs were obtained by culture system *in vitro*. **(C)** Immunofluorescence staining of OECs. GFAP: glial fibrillary acidic protein), p75: a marker for OECs.

OECs are glial cells of the primary olfactory nervous system, which are composed of the olfactory nerve and the outer nerve fiber layer of the olfactory bulb (**Figure 1**). The olfactory nerve is located between the olfactory epithelium at the top of the nasal cavity and the olfactory bulb of the anterior cranial fossa (**Figure 1**). The primary olfactory nervous system is unique in that it can constantly regenerate. Even after injury, as long as the deeper olfactory bulb inside the olfactory bulb remains intact, it can regenerate (15, 16). It is now possible to remove olfactory bulb tissue and olfactory mucosa (outermost layer and lamina propria, which belong to the central nervous system and peripheral nervous system, respectively), which also suggests the potential value of OECs therapy in central nervous system and peripheral nervous system diseases. OECs can be successfully cultured *in vitro*, which lays a foundation for the study of OECs in nerve regeneration and tissue repair (17). It is worth mentioning that OECs derived from olfactory mucosa and olfactory bulb express different characteristics, have heterogeneity, and play different roles in the repair of nerve injury (11, 18). OECs derived from olfactory mucosa can regulate the process of inflammation and the formation of extracellular matrix, but the ability of regeneration is poor. While OECs derived from olfactory bulb can promote functional recovery by inducing targeted axonal regeneration (19). Differentially expressed genes and proteins of OECs derived from olfactory bulb play a key role in nerve regeneration, axon regeneration and extension, transmission of nerve impulses and response to axonal injury. Differentially expressed genes and proteins of OECs derived from olfactory mucosa are mainly involved in inflammatory response, defense response, cytokine binding, cell migration and positive regulation of wound healing (20). All these reflect the functional differences of OECs derived from different sites.

During the development of the primary olfactory system, the axons are inaccurately located and inappropriately projected to the target layer or over-projected to the deeper layer of the olfactory bulb. Therefore, there is a large number of apoptosis of primary olfactory neurons during embryonic and postnatal development, and the axons of degenerated neurons need to be removed (21). Phagocytosis of axonal fragments in adult and postnatal animals is known to be essential for the regeneration of the primary olfactory nervous system (21, 22). Studies have shown that the phagocytosis of axonal fragments in postnatal animals. It has been found that macrophages often appear near OECs, but they only play a small role in clearing axonal fragments (23). It is considered that OECs are the primary phagocytes of primary olfactory nerve from the early stage of embryonic development (23, 24).

**Figure 2:**
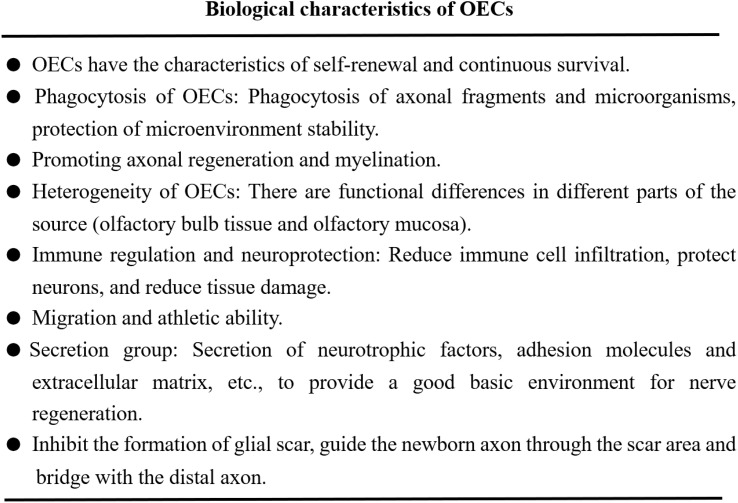
OECs play the functional characteristics of promoting nerve regeneration and tissue repair.

The regeneration and repair of neurological diseases is a complex process, involving many steps and factors. On the one hand, the key to the repair of nerve injury lies in the survival and function of neurons. On the other hand, the injured axon has the function of bridging the damaged site or the broken end (25, 26). Axon regeneration requires myelin regeneration and functional synaptic reconstruction, neurotrophic delivery, improvement of inflammation and regulation of immune response, which is essential for nerve injury repair (27). Different studies have confirmed that the functional role of OECs in nerve regeneration and tissue repair, and their biological characteristics have been recognized in the following aspects (**Figure 2**).

1. OECs can produce immune function, secrete immune regulatory molecules and exert their phagocytic activity, maintain the homeostasis of microenvironment and support neuronal survival and axonal growth (28, 29). OECs reduce the infiltration of immune cells and secondary tissue damage, protect neurons and axons in the diseased core, and help remove myelin fragments (30). 2. OECs exert their characteristics of promoting axon growth and provide structural support by extending the thin processes enclosing the axon group as insulators (22, 31). 3. OECs have the ability of migration and movement. OECs can migrate under the physiological and pathological conditions of nervous system, such as inflammation, hypoxia and neurodegenerative diseases, which are closely related to the cytoskeleton of OECs. Analysis of OECs actin cytoskeleton revealed the stress fibers, membrane spinous process, folded membrane and layered fat deposition during cell migration, as well as the distribution and migration ability of α-actin in the membrane process (32–34). 4. Phagocytic activity of OECs. OECs can remove axonal fragments and microorganisms through phenotypic changes, enhance their cytoskeleton hypertrophy and rearrangement, transition from resting state to phagocytic activity, and protect the stability of nervous system microenvironment (35, 36). Studies have shown that OECs have higher phagocytosis and transport capacity than Schwann cells and produce lower amounts of proinflammatory cytokines (37). 5. OECs can produce bioactive substances such as platelet-derived growth factor, glial-derived connexin, ciliary neurotrophic factor (CNTF), neurotrophic factor Y and cell adhesion molecules (such as L1-nerve cell adhesion molecule (L1-NCAM) and nerve cell adhesion molecule-1 (NCAM1)). These secreted bioactive substances provide a good basic environment for nerve regeneration and repair (10, 38, 39). The secretory group produced by OECs also includes paracrine exosomes and extracellular vesicles, which can protect neurons, reduce neurotoxicity and play a key role in the repair of nerve injury (40, 41). Studies have shown that OECs-derived exosomes can inhibit the polarization of pro-inflammatory macrophages/microglia, increase the number of anti-inflammatory cells, promote neuronal survival and functional recovery after spinal cord injury (42).

## 3 OECs and neurological diseases

Neurological diseases, including PD, AD, HD and ALS, are caused by disorders of protein homeostasis, characterized by the loss of specific groups of neurons and inclusion bodies consisting of insoluble and unfolded proteins. This pathogenic process leads to the gradual loss and paralysis of sensory, cognitive and motor neurons (43, 44). Although great progress has been made in the mechanisms of neurological diseases, including the identification of mutated genes that cause these diseases, but the exact mechanism of neuronal death is still unclear and there are still no effective method to slow the progression of these diseases. With the research and exploration of the treatment of neurological diseases, cell therapy has been introduced into the field of vision of researchers and has been greatly encouraged. These applied bioactive cells, including OECs, can protect neurons, reconstruct neural networks and exert therapeutic functions by producing special bioactive factors at the injured site (45–47). The goal of cell therapy in neurological diseases includes obtaining specific neuronal subtypes and rebuilding neural networks similar to those lost in the disease. Another way to treat neurological diseases is to create environmental enrichment to support host neurons by producing neurotrophic factors and removing toxic factors or by establishing auxiliary neural networks around the affected areas (48, 49). Although the application of cell therapy is still in its infancy, it has become a safe and effective new strategy for the treatment of neurological diseases.

### 3.1 OECs and Alzheimer’s disease

AD is associated with selective damage to brain areas and neural circuits critical to cognition and memory, including neurons in the neocortex, hippocampus, amygdala, basal forebrain cholinergic system and monoaminergic nucleus of the brainstem. The pathological process of AD has two typical pathological features: β-amyloid plaque deposition and hyperphosphorylated tau nerve fiber tangles (50). The existing research theory shows that the production of long A β peptide, especially in the form of highly toxic oligomer, leads to the accumulation and deposition of A β in the brain. Aggregated A β causes to neurotoxicity, degeneration of neurofibrils, activation of microglia and ultimately leads to the loss of synapses and neurons (51, 52). Diagnosis of AD is based on compliance with clinical manifestations and other auxiliary examinations, including imaging and biomarkers. The current treatment is symptomatic treatment to alleviate the progression of the disease.

OECs may have certain effects on the potential pathophysiology of nervous system diseases, including neuroplasticity and natural β-amyloid peptide. β-amyloid peptide is the main component of senile plaque peculiar to the brain of AD. The precursor protein of amyloid β protein (A β) is cut into amyloid polypeptide. Aβ 25-35 is considered to be the functional domain of A β and is responsible for its neurotoxic properties (53). *In vitro* studies found that A β may affect the molecular conformation of OECs (54, 55). After OECs were exposed to A β (1-42) fragments, the expression level of transglutaminase Tg2 in OECs increased, and the expression patterns of their subtypes (Tg2-long and Tg2-short) were also different. A β may stimulate nerve regeneration in AD by changing the conformation of Tg2 in OECs (54). Another *in vitro* study showed that A β (1-42) or A β (25-35) increased the expression of Tg2, vimentin and caspase cleavage in OECs. Basic fibroblast growth factor or glial cell line-derived neurotrophic factor can restore the protein level in OECs and play a key role in AD (56). *In vitro* studies have shown that OECs conditioned medium can improve A β (25-35)-induced oxidative damage of cells by inhibiting mitochondrial pathway, increase cell survival rate and produce protective function (53). These studies suggest the relationship between OECs and AD, but more studies are needed to confirm the usefulness of OECs as a treatment for AD.

### 3.2 OECs and Parkinson’s disease

PD caused by loss of dopaminergic neurons in the midbrain. Its pathological features are selective loss of dopaminergic neurons in substantia nigra pars compacta of ventral midbrain and ubiquitin deposition in residual neurons (57, 58). Although PD cannot be cured, there are a variety of pharmacological treatments that can alleviate some of the functional defects caused by dopamine depletion. Similarly, researchers have received consistent recognition through the application and transplantation of special bioactive cells (such as neural stem cells and mesenchymal stem cells) to treat and improve PD. In view of the biological characteristics of OECs, the treatment of transplanted OECs in PD has also achieved exciting results. Indeed, *in vivo* studies have shown that transplantation/injection of OECs into the body can improve the amount of dopamine in the striatum and restore dopaminergic activity, and play a therapeutic role in PD (59). *In vivo* studies have shown that combined transplantation of OECs and vascular endothelial cells results in a significant increase in [3H]-spirocyclone binding rate, dopamine and 3-dihydroxyphenylacetic acid levels in rats, which supports the activity of dopaminergic cells and contributes to PD-induced functional recovery (59). *In vivo* studies have shown that the transplantation of OECs into the striatum can improve the survival of neurons in the dopaminergic and inhibit the immune response of the transplantation site to improve the survival rate of striatal grafts (60). In addition, OECs can restore the nigra striatal pathway in PD and play a therapeutic role. An *in vivo* study found that OECs could improve the survival rate of grafted ventral mesencephalic tissue and promote the recovery of motor function by promoting the prolongation of dopaminergic and serotonergic axons in bridged grafts (61).

OECs express a variety of factors, such as nuclear receptor related factor 1 (Nurr1), neurogenic protein 2 (Ngn2), NGF, bFGF, GDNF, and NT3, which have neuroprotective effects on PD (59, 62, 63). *in vivo* and *in vitro* studies have shown that transplantation of OECs-Nurr1-Ngn2 can improve behavioral disorder in rats with PD (62). OECs-Nurr1-Ngn2 have significant neuroprotective, antioxidant and anti-apoptotic effects on PD by up-regulating neurotrophin-TrkB pathway (62). In the study of *in vivo* model, these growth factors and adhesion molecules expressed and secreted by OECs can also improve the survival rate of graft-derived dopaminergic neurons, facilitate communication between host and graft, and promote axonal growth (64, 65). *In vivo* studies have shown that OECs not only significantly increase the survival and neurite growth of dopaminergic neurons derived from neural stem cells, but also have a protective effect on 6-OHDA neurotoxicity under co-culture conditions (65).

Although these studies have revealed the role of OECs in the treatment of PD, but OECs transplantation has been challenged. Heterotopic transplantation of cells is unlikely to integrate into normal brain circuits. Even if OECs are transplanted into the primitive sites of PD, such as substantia nigra and striatum, new dopaminergic neurons will not regenerate the axons of striatum. Another challenge is that an obstacle to PD cell replacement therapy is the lack of abundant, reliable and renewable sources of dopaminergic neurons.

### 3.3 Cell therapy and Huntington’s disease

HD is a dominant inherited neurodegenerative disease caused by abnormal amplification of CAG repeats (36 or more) in exon 1 of the Huntington protein gene located on chromosome 4p16.3. Patients with HD mainly showed neuronal degeneration in striatum and cerebral cortex (66, 67). The most important new discovery is the highly variable nature of brain degeneration (68). The pathology of HD includes massive atrophy of the striatum and degeneration of cortical pyramidal neurons. As the development of disease, the loss of neurons becomes more comprehensive, affects many brain regions. Severe cortical loss followed, eventually leading to death (69, 70). Therefore, the use of some special graft replacement therapy to improve these striatum and neuronal degeneration can produce certain protective and therapeutic effects.

Although there are few reports on OECs in the application and treatment of HD. But some studies have found that the transplantation of functional active cells can be used as a potential treatment of HD. The therapeutic mechanisms of these transplanted cells include secreting neurotrophic factors, reducing neuroinflammation, enhancing neuronal plasticity and cell substitution. Importantly, transplanted cells (such as neural stem cells) must properly migrate to the central nervous system and integrate with host neurons to form circuits to enhance neuroplasticity (71, 72). *In vitro* studies have shown that neural stem cells/early neural progenitor cells can be cultured on a large scale and differentiated into neurons, which paves the way for the treatment of HD (73). Some *in vivo* and *in vitro* studies have found that human embryonic or fetal neural stem cells are transplanted into the striatum of HD mice or rats to survive, in some cases, improve functional status (74, 75). Despite these encouraging results, there is a lack of direct evidence and reports on OECs transplantation in the treatment of HD. Therefore, the role of OECs transplantation in HD needs more research to explore. However, cell therapy in the treatment of HD has been recognized by many studies, which is a prospective treatment strategy for the treatment of HD.

### 3.4 OECs and amyotrophic lateral sclerosis

ALS is a rapidly progressive and fatal neurodegenerative disease. In sporadic ALS, both upper motoneurons (in the cortex) and lower motoneurons (in the spinal cord) degenerate for unknown reasons. Its pathological feature is that it affects motor neurons in the motor cortex, brainstem and spinal cord, resulting in paralysis and death. Mutations were found in familial ALS cases, but these mutations accounted for only about 10% of all cases (76–78). At present, some studies have proposed several pathological mechanisms of ALS-induced motoneuron death, including glutamate-induced excitotoxicity, abnormal cytoskeleton, protein aggregation, oxidative stress, mitochondrial dysfunction and extracellular SOD1 toxicity. Currently, there is no effective treatment for this devastating disease. In recent years, researchers have proposed a therapeutic strategy of reconstructing neural function by transplanting special types of cells to replace these lost or degenerative motor neurons. For example, a clinical trial reported that mesenchymal stem cell transplantation can delay the progression of ALS and improve survival (79). *In vivo* studies have shown that transplantation of motoneurons derived from human umbilical cord mesenchymal stem cells with high expression of BDNF can improve the motor ability and prolong the survival time of mice with ALS (80). Although cell therapy for motor neuron replacement, support or as a carrier of neuroprotective molecules is in preliminary exploration and there are many challenges and problems, however, the beneficial effects of transplantation therapy observed in animal models of motor neuron disease have raised hopes (81).

The application and exploration of OECs transplantation in ALS has also achieved some exciting results. A clinical trial reported that OECs transplantation can reduce the amplitude of motor unit action potential, increase the number of motor unit action potential, and effectively control or reverse the physiological deterioration caused by ALS (82). *In vivo* studies have shown that transplantation of OECs can protect the survival of motor neurons, repair the injured axons with myelin sheath through pyramidal tract, and improve motor nerve function (83). *In vivo* studies found that OECs could prolong and increase the survival of animals with ALS by giving full play to their ability of neuroprotection and myelin regeneration (84). *In vivo* studies have shown that transplantation of OECs into the brain of rats with ALS can increase the survival and prolong the survival time of motor neurons in the motor cortex and ventral horn of the spinal cord. Behavioral tests, including screen tests, hindlimb stretching, rotating sticks and gait control, have shown that OECs transplantation can improve these symptoms (85). These studies have revealed the therapeutic role of OECs in the progression of ALS and may be used as a potential candidate cell replacement therapy for ALS therapy. But, unfortunately, people soon realized that this is an extremely difficult task because new motor neurons have to project to distant axons and connect with host neurons through neural networks to reconstruct function. The white matter in the central nervous system can produce resistance to supporting axonal growth and some evidence suggests that the toxic environment in the degenerated spinal cord may not support new transplantation substitutes, which is also the key problem to be overcome.

### 3.5 OECs transplantation and peripheral nerve injury

The process of peripheral nerve injury regeneration is a dynamic process of multicellular regulation, which involves neurons, Schwann cells and immune cells. The interaction between these cells and the molecules regulates the process of peripheral nerve regeneration. It is understood that the peripheral nervous system maintains the same inherent lack of regeneration as the central nervous system, but the difference is that after peripheral nervous system injury, neurons (which can undergo growth phenotypic changes) can produce regenerative axons. this process involves phagocytosis, clearance, secretion of factors including growth and chemokines and myelination of Schwann cells and macrophages (47). The main obstacles to the repair and regeneration of peripheral nerve injury are axonal degeneration, nerve inflammation and demyelination. Therefore, the axons that promote the repair of peripheral nerve injury mainly leads the regeneration to form a new neural connection with the axons at the distal end of the host injury, form a new neural network. Therefore, exploring and finding effective ways to change these pathological processes and make progress in a favorable direction for the repair of nerve injury. Despite the progress of microsurgical technology and the improvement of the understanding of nerve regeneration, there are still some challenges in the repair of peripheral nerve injury (86).

More and more studies have revealed that cell transplantation has been proved to play a great role in the treatment of peripheral nerve injury, which encourages people to explore the broad application and prospect of cell transplantation therapy. As described in the previous biological function of OECs, the mechanism of OECs promoting nerve regeneration is by secreting multiple neurotrophic factors, protecting neurons, inhibiting inflammatory response, and promoting axonal regeneration (10, 87). OECs can guide the establishment of new axons through the injured microenvironment to reach both ends of nerve injury and produce bridging function (26, 88). In addition, OECs transplantation has the ability to migrate and penetrate glial scar (26, 88). *In vivo* studies have shown that the transplantation of OECs effectively promotes the axon regeneration of recurrent laryngeal nerve injury and improves the function of laryngeal muscle (17). *In vivo* studies have shown that OECs are transplanted into the injured sciatic nerve, it is found that OECs can form *Ranvier* nodules of regenerated axonal myelin sheath, increase nerve conduction velocity and improve function (89). In view of the biological characteristics of OECs and the role of repairing injured nerves. Some researchers have made some achievements by combining OECs with other functional active cells or biomaterials to improve the function of OECs in peripheral nerve injury. *In vivo* experiments, the application of PCL catheter or PLGA catheter containing OECs to the site of sciatic nerve resection promoted nerve regeneration and angiogenesis (90, 91). *In vivo* and *in vitro* studies found that OECs were co-cultured with exosomes of human umbilical cord mesenchymal stem cells, these exosomes could promote the survival and migration of OECs under hypoxia, and effectively increased the gene expression and secretion of brain-derived neurotrophic factor (92). Co-transplantation of them into the injured sciatic nerve could promote the regeneration of the injured sciatic nerve and restored the motor and sensory function (92) (**Table 1**).

**Table 1:** Preclinical study on the application of OECs in neurological diseases.

Cell source	Type	Disease model	Animal	Therapeutic effect.	Refs
Olfactory mucosa or olfactory bulb	*In vivo*	Recurrent laryngeal nerve injury model	Fischer rats	OECs transplantation can effectively promote axonal regeneration of recurrent laryngeal nerve injury and improve laryngeal muscle function.	17
Olfactory bulbs	*In vivo* and *in vitro*	6-hydroxydopamine (6-OHDA)-lesioned rat model of PD	Rats	OECs can maintain dopaminergic activity in striatum, restore D-amphetamine-induced rotational behavior and spontaneous activity, and promote functional recovery in rats.	59
Olfactory bulbs	*In vivo*	PD rat model	Rats	OECs reduced the immune response (mediated by CD3+T cells and OX-6+ microglia) and increased the survival rate of dopaminergic neurons in the striatum.	60
Olfactory bulbs	*In vivo*	PD rat model	Rats	OECs can improve the survival rate of transplanted ventral mesencephalic tissue and promote the recovery of motor function. OECs can promote the prolongation of dopaminergic and serotonergic axons in bridged grafts.	61
Olfactory bulbs	*In vivo* and *in vitro*	PD rat model	SD rats	*In vitro*, it was found that OECs-Nurr1-Ngn2 increased the vitality of PC12 cells and inhibited oxidative stress and apoptosis. Transplantation of OECs-Nurr1-Ngn2/VMCs into vivo can improve the behavioral disorder of PD rats, and has significant neuroprotective, antioxidant and anti-apoptotic effects on PD.	62
Olfactory bulb tissue of SD rats	*In vivo* and *in vitro*	6-hydroxydopamine lesion rat model of PD	SD rats	OECs and transplanted fetal ventral mesencephalic tissue can enhance the functional recovery of dopamine cell survival, striatal reinnervation and rotation induced by amphetamine and apomorphine. Only in the case of combined transplantation of OECs can dopamine neurons extend to astonishingly long processes and reach the surrounding striatal septum.	64
Olfactory bulb tissue of adult rats	*In vivo* and *in vitro*	6-OHDA lesioned PD rats	Rats	OECs significantly increased the survival, neurite growth and dopamine release of dopamine neurons derived from neural stem cells, and had a protective effect on 6-OHDA neurotoxicity under co-culture conditions, promoted the recovery of motor function and neurochemistry.	65
Mouse olfactory bulb	*In vivo*	ALS mice model	Mice	The neural progenitor cells derived from the transplanted olfactory ensheathing tissue-neurospheres can differentiate into choline acetyltransferase positive large spinal cord neurons, intermediate neurons and glial cells, reduce the loss of motor neurons and promote the recovery of motor function in ALS mice.	83
olfactory bulb of neonatal “green” rats	*In vivo*	ALS rat model	SD rats	OECs transplantation can reduce the loss and collapse of motor neurons in the anterior horn of spinal cord, promote myelin regeneration and change the microenvironment of ALS.	84
olfactory bulb of approximately 7-day-old neonatal “green” male rats	*In vivo*	Adult SOD1 mutant rats with ALS	SD rats	OECs transplantation increased the survival of motor neurons in the motor cortex and ventral horn of spinal cord, prolonged survival time and behavioral tests (including screen test, hindlimb extension, rotating stick and gait control) in rats.	85
Olfactory bulbs of transgenic SD rats	*In vivo*	Rat model of sciatic nerve injury by transection	SD rats	Footprint analysis showed that OECs could survive and integrate into the repaired nerve. OECs can transform the regenerated axonal myelin sheath into *Ranvier* nodules, increase the sciatic nerve conduction velocity and improve the function of rats.	89
Allogenic neonatal rats	*In vivo*	Rat model of sciatic nerve injury by transection	F344 inbred rats	The transplantation of PCL catheter containing OECs increased the sciatic nerve conduction velocity, wet muscle weight and nerve density, and promoted the regeneration of sciatic nerve injury.	90
2 to 7 day old neonatal Wistal rats	*In vivo*	Rat model of sciatic nerve injury by transection	Wistal rats	OECs can migrate along the nerve axis after transplantation. The transplantation of PLGA catheter filled with OECs increased the nerve conduction velocity and the amplitude of compound muscle activity potential. However, 12 weeks after operation, the sciatic nerve function index was not improved due to the injury model.	91
Olfactory bulb tissue of SD rats	*In vivo* and *in vitro*	Rat model of sciatic nerve injury by transection	SD rats	Exosomes derived from umbilical cord mesenchymal stem cells can promote the vitality and proliferation of OECs and promote the motor and sensory function of injured sciatic nerve. Co-transplantation of OECs and exosomes can better promote nerve regeneration and functional recovery.	92
Olfactory bulb tissue of neonatal SD rats	*In vivo*	Rat model of sciatic nerve injury by transection	SD rats	Compared to OECs transplantation, co transplantation of SCs and OECs can increase axonal regeneration by 28%. The gastrocnemius muscle has significantly recovered and has a synergistic effect on the repair of the sciatic nerve.	93

It is understood that Schwann cells (SCs) form the myelin sheath of the peripheral nerve, protect and nourish neurons, and play an irreplaceable role in the repair of peripheral nerve injury. There is no transcriptional difference between OECs and SCs. OECs is highly similar to SCs and expresses the biomarker of SCs (94). *In vivo* studies have shown that the transplantation of OECs can regulate the changes of SCs and enhance the repair function of SCs in peripheral nerve injury, and the co-transplantation of the two can play a synergistic role in promoting nerve regeneration (93). It is worth mentioning that OECs have higher phagocytosis and transport ability and produce lower amounts of proinflammatory cytokines than SCs *in vitro*, which may be better than SCs transplantation in the damaged nervous system (37). These studies have confirmed that the functional role of OECs in peripheral nerve injury and may be used as an alternative cell therapy for peripheral nerve injury.

## 4 Clinical application and current challenges of OECs transplantation in neurological diseases

With the aging of the population and the participation of many factors, the disability and lethality of neurological diseases are increasing, but the clinical treatment of neurological diseases has always been a thorny problem. Therefore, delaying or stopping the progression of these diseases is a major but unmet urgent need. Unfortunately, due to the lack of a considerable amount of direct evidence to explain the pathological process of these diseases and to mine reliable biomarkers, this hinders the study of effective treatment and brings great challenges to clinical treatment (95). Although some drugs or other physiotherapy are used to delay or improve the progression of neurological diseases, the results are still not satisfactory or even failed. Drug therapy cannot change these pathological changes and reconstruct the neural network, which leads to the limitation of long-term clinical application.

The expected effect of OECs transplantation was confirmed in the disease model, and these studies revealed the value of OECs in neurological diseases. On the basis of the results of animal model research, some researchers have risen to the observation of clinical trials and made some progress, but most studies have focused on OECs in ALS clinical trials (**Table 2**), while other neurological diseases have not been reported. These studies suggest that OECs transplantation is safe and feasible, has therapeutic value for neurological diseases, and can improve neurological function and/or reduce progressive deterioration in patients (98, 101). OECs transplantation may play a role in the treatment of patients with ALS. A clinical trial showed that fetal OECs were transplanted into the bilateral corona radiata of the pyramidal tract of the frontal lobe in 15 patients with ALS. After 4 months of follow-up, the functional deterioration of the patients was significantly slower and the total score of ALS was increased, and the neurological function was improved in 7 cases (96). The clinical condition of 2 patients was stable, and the ALS-FRS score of the other 5 patients decreased by 4.4 points on average (96). This means that OECs transplantation seems to slow down the clinical progress of ALS. OECs were transplanted into the spinal cord and/or bilateral radiation coronary lesion areas of 327 patients with ALS and were evaluated by using the ALS functional rating scale (ALSFRS). The results showed that the spontaneous potential decreased or disappeared, the amplitude of motor unit action potential decreased, and the number of motor unit action potential increased significantly (82). Among them, 16 cases had various complications including headache, short-term fever, seizures, central nervous system infection, pneumonia, respiratory failure, urinary tract infection, heart failure and possible pulmonary embolism, and 4 cases died (82). This study suggests that OECs transplantation may effectively control or reverse the physiological deterioration caused by ALS, but it also means that there are some side effects and security risks in OECs transplantation. In other clinical trials, 7 patients with ALS received OECs transplantation, of which 2 cases showed significant improvement in short term after OECs transplantation (99).

**Table 2:** Study on OECs in ALS clinical trial.

OECs origin	Transplant method and location	Transplant dose	follow-up time	Treatment results	Side effect	Refs
Olfactory bulb tissue of human fetus	Injected into the bilateral radiation corona of the frontal pyramidal tract	Two million cells.	4 months	A total of 15 patients received OECs transplantation, the total score of ALS-FRS increased and the deterioration of neurological symptoms slowed down. Improvement of neurological function in 7 patients	No obvious side effects and complications were found, and no death after OECs transplantation was found.	96
Human embryonic OECs	Spinal cord. Radiation corona of spinal cord and bilateral frontal lobe. Double frontal lobe radiation crown	50 μl (1x10^6^) OECs was transplanted into the spinal cord, and 50 μl OECs (2 x10^6^) was injected into the radiation corona of both sides of the brain,	4 weeks	A total of 327 patients received OECs transplantation, neurological function was improved in 252 cases. In 261 cases, the spontaneous potential decreased or disappeared, the EMG amplitude decreased during contraction, and the potential density increased.	Postoperative adverse reactions occurred in 16 cases, including headache, short-term fever, major seizures, central nervous system infection, pneumonia, respiratory failure, urinary tract infection, heart failure and pulmonary embolism, of which 4 cases died.	82
Fetal OECs	Cerebral injection	2000000 of cells (100μl)	12-24 months	There are obvious glial and inflammatory reactions around the transplanted cells. There was no evidence of axonal regeneration, neuronal differentiation and myelin formation. Transplantation of OECs did not change the neuropathological changes in patients with ALS.	There was no clinical adverse reaction after operation, but the patient died of respiratory insufficiency in the later stage.	97
Fetal OECs	Corona radiata	100μl (about 2 million)	An average of 47.2 months	OECs transplantation could not prevent the progression of disease in patients with ALS.	No obvious adverse events were observed.	98
Olfactory ensheathing tissue of female fetus born at 12-16 weeks	Brain parenchyma	100μl (about 2000000)	2 weeks	Seven patients with ALS were treated with OECs transplantation, of which 2 patients’ ALSFRS score increased and their neurological symptoms and EMG were improved. OECs improved the symptoms of the patients in a short time. and the other 5 patients remained stable.	No obvious adverse reactions were reported.	99
Embryonic olfactory bulb tissue	Unreported		1 year	Symptoms of 7 patients were not improved objectively after receiving OECs treatment. OECs had no beneficial effect on the treatment of patients with ALS.	Two patients developed venous thrombosis and respiratory insufficiency	100
Embryonic olfactory bulb tissue	Spinal cord and brain	Unreported	2-8 weeks	Transplantation of OECs into the brain and spinal cord is feasible and safe. They have good therapeutic value for central nervous system diseases such as chronic spinal cord injury, ALS, cerebral palsy and stroke sequelae, and can improve patients’ neurological function and/or reduce progressive deterioration.	Among the 1255 patients with ALS, 4 patients died of heart failure, pulmonary embolism, respiratory failure and severe pneumonia.	101

However, OECs transplantation is not very satisfactory in human trials of neurological diseases, and it has always been controversial. Some studies have found that OECs transplantation is not beneficial to patients with ALS (100). A case report showed that female patients with ALS who received frontal lobe injection of OECs, found that ALS progressed faster after surgery and suffered crippling side effects (102). Although this case shows that OECs transplantation has achieved poor results in the treatment of ALS, this is only a case and does not represent the feasibility of OECs transplantation treatment. More case data are needed to support this result. Another clinical trial found no evidence of axonal regeneration, neuronal differentiation and myelin formation after transplantation of fetal OECs (97). It was further found that the transplanted cells did not change the neuropathological changes in patients with ALS (97). There are many factors leading to these results, which may be related to the differences in symptoms and signs of patients, time of nerve injury, operation, time and dose of OECs transplantation and evaluation index.

Although animal experiments have recognized the value of OECs transplantation in the treatment of neurological diseases, the inherent variability and diversity of human patients have been ignored. Therefore, it is not clear whether these transplanted cells will produce similar results when used in patient populations, and there is a lack of more direct data support in clinical trials. At this stage, it is not feasible to extrapolate the results of these *in vivo* studies directly to human patients. Therefore, the results and functional effects of transplanted OECs in animal experiments need to be verified in clinical trials. But at present, the application of OECs in clinical trials is greatly restricted, this may be due to some problems that need to be solved to hinder its development and application in clinical trials.

The first problem is the stability and safety of transplanted OECs. Studies have shown that transplantation of OECs into the body can produce some side effects, such as headache, short-term fever, seizures and even aggravate the progression of the disease (96, 102). Due to the differences between animals and humans, the active therapeutic effects of OECs in animals and produce favorable results, but these may not be significant in humans or even the opposite results. A major obstacle to the progress of OECs therapy is to understand how OECs work in the body and how they integrate with the target tissue/organ to overcome tissue and environment-specific barriers. Therefore, the extensive use of OECs in clinical trials requires more animal research to fully understand the characteristics and safety of OECs therapy and reduce the risk of additional injury.

The second major problem is that OECs can be derived from olfactory mucosa or olfactory bulb tissue. however, it takes a long time to prepare OECs, and the purification system of OECs *in vitro* is still not perfect, which may miss the key treatment window. Porcine OECs changed systematically with time *in vitro*, resulting in decreased expression of p75, decreased proliferation and decreased myelin regeneration ability (103). This means that it takes a relatively short time for the expanded culture and purification of cells *in vitro* to enhance the viability and proliferation of cultured cells. Therefore, it is very important to obtain safe and efficient cells in a short time. However, the technology and instruments currently used are difficult to achieve, although the task is arduous, but with the continuous efforts of researchers will eventually be solved.

The third problem is that transplantation of autologous or allogeneic OECs into the body will produce a certain degree of immune rejection, which can reduce the success rate and survival rate of cell transplantation, reduce the therapeutic effect and even cause immune injury. The microencapsulated cell technology has good biocompatibility, can exert immune barrier and anti-inflammatory effects, increase the survival rate of transplanted cells, and contribute to the therapeutic effect of transplanted cells in the field of treatment (104–106). We previously explored the effect of transplantation of microencapsulated OECs into sciatic nerve injury in pain relief. The results showed that compared with OECs alone, microencapsulated OECs could bitter promote the myelination of sciatic nerve and relieve pain (107, 108). Although this technique has been recognized by researchers and feasible in the basic field, but clinical trials based on cell encapsulation have not produced any approved treatments. Progress in this area has been slow, in part because of the complexity of technology and possible uncertain risk factors such as biodegradability, toxicity and metabolism. Therefore, exploring a safe and feasible method to reduce the immune response and inflammatory response of OECs transplantation into the host, and improve the survival rate and survival time may improve the therapeutic effect.

The fourth problem is that the methods of OECs transplantation into the body including intracranial injection, intramedullary injection and intravenous injection. Different transplantation methods will produce different therapeutic effects. At present, there is no tracking method to determine whether the transplanted cells can accurately locate and gather in the injured site, especially for vein transplantation or subarachnoid transplantation, the cells will disperse and move in the blood or brain effusion. It is possible that only a few transplanted cells will be colonized in the trauma site, which will significantly reduce the therapeutic effect and increase other additional potential risks. We believe that using image guidance, puncture or minimally invasive surgery to accurately implant cells into the injured site or the site that needs treatment may be the best solution at present. In addition, different doses and time of transplantation of OECs have different effects. At present, there is no unified application standard for the best time and quantity of cell transplantation.

The fifth challenge is that for peripheral neurodegenerative diseases, OECs transplantation can protect injured neurons, promote axonal regeneration and repair injured nerves by secreting neurotrophic factors. However, these factors such as BDNF and NGF can also be used as pain-related molecules (11, 47). It may lead to pathological pain in the process of repairing injured nerve. Therefore, it is necessary to fully understand the characteristics of transplanted cells and the specific mechanism by which transplanted cells mediate secretory factors to regulate nerve injury.

Although cell transplantation is a promising choice in current and future clinical applications, the natural limitations of cell source and *in vitro* expansion are still the main challenges to the development of autologous cell transplantation. This difficulty may be overcome by immortalizing primary cells, allowing unlimited expansion and reprogramming of somatic cells to produce a large number of induced pluripotent stem cells. Although cell transplantation therapy is in the primary stage of exploration, there are many uncertain factors and many problems that need to be solved, but these will not prevent researchers from carrying out in-depth exploration and intensified work.

## 5 Conclusion

The pathological mechanism of neurological diseases is complex, and treatment has always been a thorny problem at present, but the clinical effect of traditional medicine is not good, and even has devastating sequelae. The development of a cell-based therapy for the treatment of neurological diseases is a prospective exploratory method. As many studies have shown, considerable progress has been made in the direction of cell therapy. OECs are characterized by their unique biological characteristics, lifelong survival and continuous renewal, and play a favorable role in nerve regeneration and tissue repair. The possible mechanisms of OECs in the treatment of neurological diseases include the secretion of neurotrophic factors, immune regulation, axonal regeneration, myelination and neuroprotection, thus improving and delaying the progress of neurological diseases. However, the results obtained in clinical trials are not very satisfactory, and the effectiveness of these cell-based therapies remains to be proved by more studies. Before OECs may be used in clinical trials to treat neurological diseases, many existing problems need to be solved, and more in-depth studies on the practical application of cell therapy strategies are needed. Despite these unresolved issues, with the continuous efforts of researchers, the cell transplantation strategy is expected to be successfully applied to clinical treatment in the future.

## Author contributions

L-PZ: Data curation, Formal analysis, Methodology, Writing – review & editing. J-XL: Data curation, Formal analysis, Writing – review & editing. Y-YL: Investigation, Methodology, Software, Writing – review & editing. H-LL: Supervision, Writing – review & editing. W-JZ: Conceptualization, Funding acquisition, Writing – original draft, Writing – review & editing.
